# Phenological Development of Waxy-Leaved Mustard (*Boreava orientalis* Jaub. and Spach.)

**DOI:** 10.3390/plants15050700

**Published:** 2026-02-26

**Authors:** Taiebeh Adeli, Iraj Tahmasebi, Sirwan Babaei, Christian Andreasen

**Affiliations:** 1 Department of Plant and Environmental Sciences, University of Copenhagen, Højbakkegaard Alle 13, 2630 Taastrup, Denmark; taiebeh@plen.ku.dk; 2 Department of Crop Production and Genetic, Faculty of Agriculture, University of Kurdistan, Pasdaran Boulevard, Sanandaj 15175-66177, Iran; i.tahmasebi@ac.ir (I.T.); s.babaei@ac.ir (S.B.)

**Keywords:** base temperature, BBCH scale, eco-biology, GDD, invasive weeds

## Abstract

Waxy-leaved mustard (*Boreava orientalis* Jaub. and Spach.) is an invasive weed that has rapidly spread across wheat fields in the Kurdistan Province, Iran. The germination and phenology of this species were studied through a series of greenhouse and field experiments conducted from 2018 to 2020 to better understand its biology and support effective management strategies. We calculated the growing degree days (GDD) required for each growth stage of *B. orientalis* and related the calculations to the Biologische Bundesanstalt, Bundessortenamt und Chemische Industrie (BBCH) scale. We also studied whether light affected germination. The results indicated that light significantly reduced germination. The base temperature for germination (4 °C) is identical to that of wheat, and the growth periods were largely similar. Consequently, the maturation of wheat and *B. orientalis* seeds co-occurred, leading to the dispersal of weed seeds during wheat harvest and increasing field infestation. Understanding the phenological development of *B. orientalis* provides a valuable basis for developing management strategies and implementing effective control measures to reduce field contamination and prevent further spread.

## 1. Introduction

Invasive plants present significant ecological and economic challenges [1]. Among these, invasive species are particularly critical due to their rapid spread and ability to establish in new areas, often without human intervention. Such species can result in substantial environmental, economic, and health-related impacts [2]. In recent years, their uncontrolled expansion has become a significant threat to global ecosystem diversity, agricultural productivity, and ecological function [3]. Factors such as climate change, international trade, and socio-economic shifts will further accelerate plant invasions and intensify their effects on native biodiversity [4]. Iran’s unique geography and diverse topography give rise to a wide range of climatic zones, from arid deserts to dense forests, supporting high biodiversity. Recent assessments indicate that 311 alien plant species have been documented in Iran, of which 13 are currently recognized. Observations underscore the urgent need for ongoing monitoring and proactive management [5]. Ecosystems are increasingly threatened by invasive plants, which can out-compete native species and disrupt ecological balance [6].

Weak biosecurity measures have further exacerbated this issue, facilitating the establishment and spread of invasive species across regions. Given Iran’s distinctive agricultural, environmental, and economic conditions, understanding the traits that contribute to weed invasiveness is critical.

*Boreava orientalis* is an invasive Brassicaceae species found in wheat fields of Kurdistan Province, western Iran [7]. This dicot exhibits hypogeal germination and is classified as invasive due to its establishment outside its native range, ease of identification, and rapid spread [8]. Invasive and non-invasive species differ in key traits, including physiology, morphology, biomass accumulation, and growth rate. For instance, high photosynthetic rates, large specific leaf area, low root-to-shoot ratios, high fecundity, and rapid relative growth rate are often associated with invasiveness [9]. Species that exhibit high dry matter production and competitive canopy ability are more likely to survive and thrive in diverse environments [10]. Understanding the ecobiology of a weed requires detailed knowledge of its phenology, which is essential for effective management and control.

Phenology refers to the relationship between climatic factors and successive events in a plant’s life cycle [11]. Some researchers define phenology as the study of periodic biological events occurring at the organ, tissue, or cellular level [12]. Environmental factors such as temperature and light significantly influence plant phenology and growth rate [13]. Flowering phenology is a critical determinant of a plant’s invasive potential [9]. Phenology plays a key role in determining the outcome of weed–crop competition, with temperature and light being the most crucial factors driving development [14]. Plants’ evolutionary adaptation and phenotypic flexibility determine their capacity to flower and set seeds in new environments [15]. Subsequently, species-specific scales have been designed to capture detailed growth characteristics of individual crops [16].

Several scales have been developed to study plant phenology, among which the BBCH scale (Biologische Bundesanstalt, Bundessortenamt und Chemische Industrie) [17] is widely recognized as a standardized, decimal-based system describing plant growth stages. It provides unified terminology that supports international agricultural research and promotes interdisciplinary communication. In 1989, Bleiholder et al. introduced a general BBCH decimal coding system to describe the growth stages of crops and weeds, building on the decimal scale for cereals and rice developed [18]. In 1992, Hack and colleagues proposed the extended BBCH scale, which introduced a more precise and comprehensive coding system. This version organizes plant development into ten principal growth stages, each subdivided into secondary stages, allowing detailed characterization from germination to senescence. The extended BBCH scale has since been widely adapted to create species-specific scales, providing accurate and detailed descriptions of developmental stages [17]. Each BBCH development stage transition (e.g., from leaf development to stem elongation, or from flowering to fruit development) often corresponds to a certain amount of accumulated (Growing Degree Days) GDD (i.e., heat units). For example, a crop might need 700 GDD to go from BBCH stage 51 (flower buds visible) to BBCH stage 65 (full flowering). Thus, one can estimate, after sowing (or after a defined starting point), that once the accumulated GDD reaches threshold X, the crop will reach BBCH stage Y. This enables scheduling of interventions (pesticides, fertilizer, irrigation) timed by GDD rather than calendar days [19].

In practice, the BBCH scale and GDD complement each other. BBCH provides the growth stage the plant is in (qualitative/ordinal), while GDD provides a quantitative time/heat BBCH scale and calculated GDD to identify growth stage timings. They reported, for example, that flowering (BBCH 63) began at 633–809 GDD depending on the year [20]. Some research uses GDD as the independent variable, BBCH stage as the dependent variable, and cumulative GDD to determine the number of heat units required for each stage transition [21].

We studied the phenological growth stages of *B. orientalis* using GDD and the extended BBCH scale, providing a practical framework for research on this invasive weed species. The BBCH coding system for identifying plant growth stages offers an opportunity to describe plant development non-destructively and independently of environmental influences. This coding system serves as an effective communication and management tool, enabling the organization and coordination of agricultural practices in line with integrated plant management strategies, including crop protection. This study aims to investigate the phenology of *B. orientalis* using the BBCH scale to assess its ecological and agricultural implications and to inform sustainable management strategies to limit its spread. Phenology-based approaches use temperature, moisture, and day-length models to predict weed emergence waves, peak periods of competition, and seed shattering periods, which is important for seed bank management and modeling seed rain, to help schedule late-season weed control to prevent the seed bank from increasing. We hypothesize that the development of *B. orientalis* and wheat happens simultaneously, which could be the reason for its rapid spread in wheat fields in Iran.

## 2. Results

### 2.1. Laboratory Experiment

#### The Effect of Light on Germination

**Figure 1:**
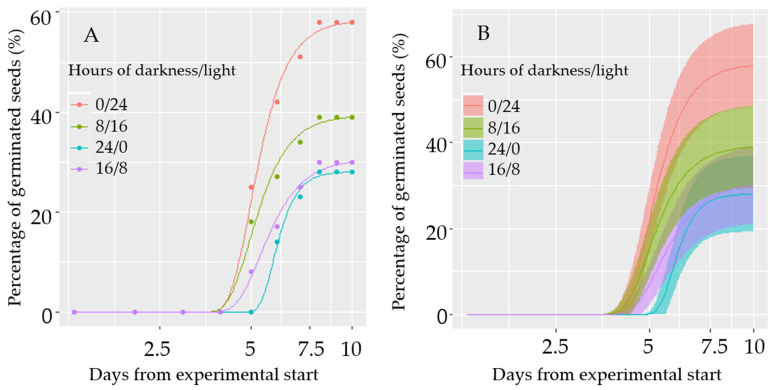
(**A**) The effect of light on germination of *B. orientalis.* (**B**) With confidence bands.

Light had a significant adverse effect on germination (*p* < 0.05) (Figure 1A). The highest germination percentage (14.5%) was under complete darkness (0/24), and the lowest germination percentage (7%) was in light (24/0). The effect of light on germination of *B. orientalis* with confidence bands showed that the light condition overlaps with 8 D/16 L, and the darkness condition overlaps with 8 L/16 D (Figure 1B).

### 2.2. Greenhouse Experiments

#### 2.2.1. Phenological Development of *B. orientalis*

Fifty percent of the seeds emerged in the greenhouse on 28 September. The first leaves appeared on 7 October in 50% of the pots. The second leaf appeared on 10 October in 50% of the pots. On 18 October, four permanent leaves appeared in 50% of the pots. On 25 October, the plant stems were visible in 50% of the pots. On 2 November, flowers appeared in 50% of the pots, and during the first 10 days of November, pollination occurred. Seed development began in the last 10 days of November. In early December, the seeds were completely dried and matured. The phenology stages of *B. orientalis* are shown in Table 1 and Table 2, respectively. Germination started on 23 March and took two weeks. The seeds required a cold period to break the dormancy. The seeds ripened at the same time as the wheat (about 10 December). Seed production lasted 30 days and ended after 1261 GDD. The development of the stem was the shortest stage, lasting 5 days and ending after 46 GDD. The appearance of primary leaves occurred after 270.3 GDD, followed by stem extension, which started at 46 GDD. In total, it took about 2624 GDD to complete the plant life cycle and ripen the seeds.

Physiologically, 0 °C is the lowest temperature for plant growth. The base temperature for long-day plants is between 0 and 5 °C [22]. The base temperature of *B. orientalis* and wheat is 4 °C [23]. The appearance of the primary leaves and the stem elongation of *B. orientalis* and the wheat cultivar co-occurred. Flowers developed on *B. orientalis* from 5 May, while wheat completed flowering on 12 May. The seed production of *B. orientalis* began on 15 June and finished on 26 June. Wheat spikes developed from 21 May, and all seeds matured on 21 June. During wheat harvesting, *B. orientalis* shattered its seeds and increased the soil seed bank.

#### 2.2.2. Phenological Development of *B. orientalis* in Greenhouse According to the BBCH Scale

- Principal Stage 0: Germination/Seedling

00: Dry seed

Mature seeds of *B. orientalis* uptake water but exhibit dormancy due to a hard seed coat. Seed dormancy is primarily physical, preventing water from entering the seed and delaying germination until environmental conditions are favorable, ensuring that seeds germinate only under optimal conditions, which contributes to the weed’s persistence and invasiveness in wheat fields.

01: Beginning of seed imbibition

When a mature, dry *B. orientalis* seed is placed in a moist environment, it begins to absorb water through the seed coat, initiating metabolic activity.

03: Seed swelling

The seed coat softens and enlarges due to water absorption. This process transitions the seed from dormancy or quiescence into active germination phases.

05: Radicle emergence

The embryonic root emerges, anchoring the seedling and initiating nutrient uptake. In *B. orientalis*, radicle emergence occurs after 4 days when seeds have imbibed and dormancy is broken, facilitating early establishment in competitive wheat fields.

07: Hypocotyl emergence

The hypocotyl elongates, pushing the cotyledons above the soil surface. *Boreava orientalis* exhibits hypogeal germination. Then, photosynthesis immediately provides energy for further seedling development.

09: Emergence of seedling

Seedling breaks the soil surface; cotyledons may be visible and start photosynthesis. cotyledons emerge quickly once conditions are favorable, and they begin photosynthesis soon after.

10: First leaf

The first true leaf fully opens, starting active photosynthesis. In *B. orientalis*, like other small-seeded annuals, this rapid onset of photosynthesis is vital, since seed reserves are limited. Within a few days, true leaves emerge from the shoot apex, and the plant transitions to independent autotrophic growth.

11: First leaf fully expanded

Leaf reaches maximum size, and photosynthetic capacity increases. In the *B. orientalis*, often with glaucous and waxy leaves.

12: Two leaves

The second leaf emerges and expands, and energy production rises. At BBCH stage 12, the plant has two fully expanded true leaves. This is an important early vegetative growth phase in which energy production increases rapidly. The second true leaf fully emerged. The second leaf has fully unfolded and widened. The photosynthetic area nearly doubled compared to BBCH 11.

13: Three leaves

The third leaf emerges, supporting plant growth. The third leaf develops from the shoot apical meristem. It typically appears alternate, as the phyllotaxy shifts from alternate cotyledons to a spiral pattern common in Brassicaceae. This third leaf is usually larger, with a more defined petiole and blade, and often shows the characteristic waxy texture of the species.

19: Nine or more leaves

Full vegetative leaf canopy established, maximum photosynthetic activity. The plant now carries a full rosette or small, branched canopy of leaves. Leaf area is at its maximum.

- Principal Stage 3: Stem elongation/vegetative growth

30: Beginning of stem elongation

Internodes begin elongating, and the plant gains height. During early vegetative growth, *B. orientalis* maintains short internodes, forming a low rosette of overlapping leaves close to the soil surface.

31: 1st internode elongation

The space between the first and second leaves increases. In many Brassicaceae weeds [24], including *B. orientalis,* early seedling development is described in terms of leaf appearance and internode elongation.

32: Second internode elongation

The second internode grows, elongating the stem. In phenology, this corresponds to the stem elongation phase in the BBCH scale, when new internodes become visibly extended. Although this is a generic pattern, it is not specific to *B. orientalis*.

39: Stem elongation complete

Stem has reached its mature vegetative length and is ready for reproductive development. The main stem of *B. orientalis* is fully elongated and reaches its maximum vegetative height. The plant now displays distinct nodes and internodes, with leaves arranged alternately. The plant transitions from a compact rosette (basal leaves) into a fully extended flowering stem. *Boreava orientalis* reaches a height of about 20–30 cm at full vegetative elongation, consistent with field observations in wheat fields (7; 43). Internodes lengthen rapidly, lifting the inflorescence zone upward and helping future flowers disperse seeds over a wider radius.

- Principal Stage 5: Inflorescence Emergence

50: Flower buds visible

Small floral buds appear on shoot tips. The first small yellow floral buds appear at the shoot tips, marking the transition from vegetative to reproductive growth. The shoot apex differentiates into a floral meristem, and leaf production slows because this weed has determinate growth. The shoot apical meristem switches from vegetative to reproductive. Tiny yellow-green floral buds appear on the branches and the top of the main stem. Leaf production slows significantly because *B. orientalis* shows determinate inflorescence development, like many small annual Brassicaceae. Internodes continue to lengthen slightly, but at a much slower rate than in stage 39.

51: Flower buds swelling

Buds enlarge; petals start forming internally. Buds enlarge as internal floral organs, sepals, petals, stamens, and pistil form. The outer sepals protect the developing petals. Stem elongation continues slowly.

55: Flower buds

Petals visible but unopened, reproductive organs developing. Petals become visible within the closed buds, showing the characteristic four-petal cruciform shape of Brassicaceae. Reproductive organs mature in preparation for fertilization. Yellow petals are now visible through the partly separated sepals. The flower is still closed, but the plant is days away from openinguntil full flowering.

59: First flowers open

Initial flowers bloom, starting the reproductive phase. The earliest buds open, initiating the reproductive phase. The plant now begins active pollination, often facilitated. This stage refers to the point when the first flower on the main shoot begins to open. In phenological scales such as the BBCH scale, this is often marked as a transition from vegetative growth to reproductive (flowering) growth. The opening of the first flowers signals that the plant has committed resources to reproduction; it will begin pollination, set fruit (pods), and eventually produce seeds. For a weed like *B. orientalis,* assuming it behaves similarly to other Brassicaceae, this is a critical stage; the plant is mature enough to reproduce, and removal or control at or just before this point can reduce seed set and future weed pressure.

- Principal Stage 6: Flowering

60: Beginning of flowering

Flowering begins on multiple buds. Several buds open simultaneously on the terminal raceme. Flowers display four yellowish petals. The first open mustard-type flowers (4 petals, typical crucifer form) appear on some plants in the population. Immediately after BBCH 60, more flowers open progressively (BBCH 61–65)

61: 10% of flowers open

Early bloom stage: the first round of pollination begins. Active pollen transfer ensures genetic variation. At 10% open, there are already a substantial number of receptive flowers, indicating that active pollen transfer can start. This is a critical period for mating, especially in cross-pollinated species.

65: Full flowering

Most flowers are open during the peak pollination period. Most flowers are open across the raceme. This is the peak reproductive period, with maximum pollination activity and fertilization. The plant maintains high photosynthetic activity to support the development of reproductive organs. This weed requires vernalisation (cold requirement) for flowering, which influences when it reaches its reproductive (flowering) phases under field conditions. Its flowering time is highly variable and can adapt under selection. At BBCH 65, *B. orientalis* would likely present half of its potential flowers open, forming a dense floral display on many raceme branches, optimizing for pollination and seed set.

69: End of flowering

Flowering concludes, petals begin to fall, and fertilization occurs. Most petals begin to wither and fall. Fertilized ovaries expand into young green pods. The plant’s energy shifts toward fruit and seed formation. Most of its yellow mustard-type flowers have already bloomed, and many of the petals are falling or drying. The plant will enter its reproductive phase, with many fertilized flowers now developing into siliques (seed pods). Each pod contains only one seed. As pods develop, the plant’s main investment goes into seed maturation.

- Principal Stage 7: Fruit Development

70: Fruit set begins

Fertilized flowers start developing into fruits. Newly fertilized ovaries enlarge to form small green pods. Its inflorescences have many flowered racemes, and in the fruit, the pedicels can be up to 5–7 mm long. It is a one-seeded, beaked, ovate (egg-shaped), about 8–10 mm long, possibly with small bumps and one seed between the wings. Thus, at fruit set, the fertilized ovary enlarges into this type of pod, and the embryo inside the seed begins to develop.

71: Fruit 10% of final size

Young fruits are small and visible. Pods become clearly visible and start elongating. Embryo and endosperm tissues differentiate. Young fruits are small, green, and elongating on the racemes. The pedicel supporting each pod is already 5–7 mm.

75: Fruit 50% of final size

Fruits reach half-maturity; seeds are developing inside. Fruits expand rapidly. Seed coats thicken, chlorophyll begins to decline, and internal moisture decreases. *Boreava orientalis* produces indehiscent, ovate pods. At BBCH 75, pods are approximately half their final length (4–5 mm). Seeds inside pods are differentiating further, with endosperm and embryo tissues maturing, and the seed coat beginning to harden. The pod wall begins chlorophyll degradation, turning from green to pale green as seeds accumulate storage reserves.

At BBCH 79, pods have reached nearly full size, are firm, and have lost most chlorophyll. Fruits reach almost full size and weight; physiological maturation is progressing. Pods reach their final shape and size. Seeds harden and darken, indicating physiological maturity. The plant starts diverting resources from leaves to seeds. By BBCH 79, seeds are nearly mature; interventions at this stage may still prevent seed dispersal if pods are removed. However, once seeds reach full maturity (BBCH 89), control becomes less effective because seeds are ready for soil storage.

- Principal Stage 8: Ripening

80: Beginning of ripening

Fruit color changes. The color of the pod changes from green to pale yellow or brown as chlorophyll breaks down. The pods begin to dry. At BBCH 80, pods begin color change from green to pale yellow, signaling the start of ripening.

81: 10% of fruits ripe, 85: 50% of fruits ripe, 89: Full ripeness

A small proportion of the fruits are fully mature. A small number of pods are completely dry and contain viable seeds. Half of the fruit is fully ripened. Half of the fruits have reached full maturity, and seeds show high viability and dormancy potential. All fruits are mature. All pods are dry. Seeds are dispersed by shattering or wind, entering the soil seed bank for the next growing season. Given that *B. orientalis* is an annual and reported in agricultural fields, it likely follows a pattern where a subset of pods matures early (10%), then more pods ripen (50%), and eventually many or all pods become dry. The dehiscence would release viable seeds into the soil. This is consistent with many Brassicaceae family weeds [7].

- Principal Stage 9: Senescence

90: Beginning of leaf yellowing, 91: 10% of leaves yellow, 95: 50% of leaves yellow, 99: Plant dead or dormant.

Leaves start losing chlorophyll. Lower leaves begin to yellow due to nutrient remobilization toward the seeds. Photosynthetic rate declines. Early leaf senescence, nutrient reallocation begins. The process of senescence starts visibly; the plant begins to dry from the base upward. Significant leaf drop: plant prepares for dormancy. Most leaves have lost chlorophyll. The plant’s growth ceases, and only the fruits remain functional for final seed drying. Complete senescence, the plant enters dormancy until the next growth cycle. The entire plant dries and dies. Mature seeds remain in the soil, ensuring regeneration in the next favorable season, a key survival strategy for annual weeds like *B. orientalis*. By 50% leaf yellowing (stage 95), much of the plant’s function likely ceased. By stage 99, the plant may be fully dead or dormant, with above-ground biomass dried. The dead plant then makes way for seed dispersal into the soil seed bank (Table 1).

#### 2.2.3. Phenological Development of *B. orientalis* in the Field

**Figure 2:**
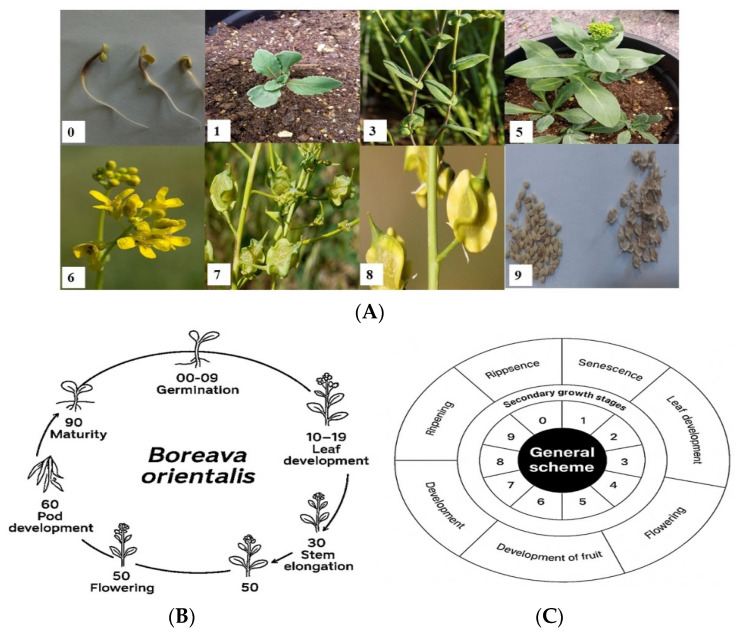
*Boreava orientalis’* phenology stages according to the BBCH scale. (**A**) Growth stages of *B. orientalis* according to the BBCH scale. (**B**) Life cycle of *B. orientalis* according to the BBCH scale. (**C**) Scheme of phenology stages of *B. orientalis* according to the BBCH scale.

Cold conditions induce seed dormancy. After the cold season, it begins to grow by accumulating the necessary GDD and shows rapid growth in a short period. On 23 December, germination was observed in 50% of the plots, and cotyledons emerged from the soil. The growth stopped completely during the winter. Two permanent leaves appeared on 22 April in 50% of the plots. The leaves were blue-green and had a smoother, waxy surface than the cotyledons. On 27 April, stem elongation was recorded in 50% of the plots. The stems’ surface was also waxy. Flowering started in 50% of plots on 5 May. All plants flowered on 15 May. From 15 June, seeds developed, and all were completely ripened on 26 June (Figure 2A). The phenological development of *B. orientalis* in the field and the GDD from planting to ripening are shown in Table 2. The life cycle and scheme of *B. orientalis* according to the BBCH scale is shown in Figure 2B,C.

## 3. Discussion

### 3.1. Laboratory Experiment

#### Light Effect on Germination of *B. orientalis*

Light had a significant adverse effect on seed germination. The highest germination percentage (14.5%) was under complete darkness, and the lowest germination percentage (7%) was in 24 h of light, indicating that this species can germinate under a wide range of light conditions. However, for many other weed species, light plays a crucial role in breaking dormancy and initiating germination, highlighting interspecific differences in germination ecology. The growth and phenology of plants under a canopy or in competition with other plants may differ due to variations in light quantity and quality, compared to weeds growing without direct plant competition. The effects of light quantity and quality have been demonstrated for several weed species, such as redroot pigweed (*Amaranthus retroflexus* L.) [25]. In a related long-day species, wild mustard (*Sinapis arvensis* L.), the number of days to complete its life cycle is lower under long photoperiods [24].

As light did not trigger germination, the release of seed dormancy after the winter may reduce the potential establishment of new plants and the soil seed bank. When seeds germinate from deeper soil depths, they may not have enough energy stored to ensure that the cotyledons reach the soil surface and are able to establish seedlings, and, therefore, die.

### 3.2. Greenhouse Experiments

#### 3.2.1. Phenological Stages of *B. orientalis* Related to GDD

The phenological development of *B. orientalis* is strongly influenced by accumulated heat, quantified as GDD, which provides a precise measure of thermal time required for the transition between developmental stages, from germination to seed maturation, and allows prediction of critical growth events under varying environmental conditions. Understanding GDD thresholds for *B. orientalis* is essential for synchronizing management interventions.

A study of the life cycles of *Setaria viridis* (L.) P. Beauv., *Echinochloa crus-galli* (L.) P. Beauv., *Galium parine* L., *Solanum nigrum* L., *Cynodon dactylon* (L.) Pers., and *Ranuculus* sp. showed that temperature was the most important ecological factor for phenological development [15]. Such studies are useful for predicting weed growth and can determine the appropriate time to use mechanical removal or herbicide application, at the weed’s most vulnerable stage.

#### 3.2.2. Phenological Stages of *B. orientalis* Related to the BBCH Scale

Emergence (BBCH 00–09): Typically begins at low GDD thresholds, marking the transition from imbibition to visible seedling emergence. In a few species with hard seed coats, water can enter the seed, but the hard seed physically restricts the growth of the embryo. Most species with hard seed coats maintain dormancy by preventing the entry of water into the seed [26].

Several weeds produce seeds with hard, impermeable seed coats (e.g., *Abutilon theophrasti* Medik.), which make water absorption difficult, and keep them dormant until the coat softens or cracks [27].

- Leaf Development (BBCH 10–19): during which the plant establishes its photosynthetic capacity.
- Many Brassicaceae weeds (e.g., *S. arvensis*) have shallow emergence, light-dependent germination, and persistent seed banks [24]. It ensures quick canopy establishment. Early photosynthesis helps the weed outcompete crops for light and nutrients. Stem Elongation (BBCH 30–39): rapid vertical growth begins.

In *B. orientalis*, stem elongation starts shortly after the expansion of the first true leaves. This species produces a tall, competitive flowering stem, allowing it to overtop wheat plants later in the season. Stem elongation in *B. orientalis* progresses steadily with increasing GDD.

- Bolting and Pre-Flowering (BBCH 50–59).

In *B. orientalis*, bolting also begins shortly after the formation of several true leaves, and the plant quickly transitions from a rosette-like juvenile stage to an upright architecture. The onset of bolting is strongly correlated with GDD accumulation and is one of the earliest indicators that reproductive development is imminent.

- Flowering (BBCH 60–69)

For *B. orientalis*, flowering begins relatively early in the season and is prolonged, allowing the species to produce viable seeds over an extended period. Flowering typically overlaps with sensitive wheat growth stages, which increases resource competition. Although *B. orientalis* is primarily self-pollinating, partial outcrossing may occur, helping maintain genetic diversity and adaptability across different environments. peak reproductive activity and maximum cross-pollination potential occur.

- Pod and Seed Development (BBCH 70–79).

In *B. orientalis*, once pods are formed, the plant rapidly enters a seed-filling phase that is highly efficient even under competitive field conditions. Pods are small, but a large number are produced, resulting in more than 50 seeds per plant. Even when plants are damaged or partially defoliated, they can still form viable seeds due to strong reproductive plasticity.

Failure to control *B. orientalis* before this phase results in substantial seed bank replenishment, contributing to long-term field infestation. Because seeds mature progressively from lower to upper pods, late-season management often fails to prevent the addition of seeds to the soil. At BBCH 69, the weed still produces many seeds. However, removal or treatment after this stage may miss earlier set pods and may not prevent full seed bank replenishment [28].

The pod wall begins chlorophyll degradation, turning from green to pale green as seeds mature. The pod wall begins chlorophyll degradation, turning from green to pale green as seeds accumulate storage reserves. Embryo differentiation begins, and endosperm forms as nutritive tissue for the seed. Early cellular differentiation at BBCH 71 determines potential seed viability and size [29].

### 3.3. Field Experiments

In the *B. orientalis* field experiments, the plants developed slightly faster (BBCH 10–14) and showed a more stable leaf expansion during short periods of evaporation stress.

Bolting (BBCH 50–59) generally aligns with similar GDD thresholds as other Brassica, though stem elongation progresses more rapidly once initiated. GDD-based scheduling improves timing of nitrogen application (BBCH 30–31), monitoring before BBCH 12–14,and weed control prior to BBCH 14 when canopy cover is still limited. Overall, integrating BBCH staging with GDD models strengthens predictive accuracy and supports more effective field management of *B. orientalis.* Combining both allows highly accurate phenology tracking, and GDD predicts when a plant transitions to a BBCH stage. GDD models help with ensuring proper timing for agronomic operations. Weed control is most effective when synced with early crop stages (BBCH 10–16).

### 3.4. Greenhouse vs. Field Phenology

The development of *B. orientalis* deviated significantly between the greenhouse and the field. While the growing conditions were stable in the greenhouse (*e.g*., uniform soil, controlled water, light, and fertilizer supply), the growing conditions fluctuated significantly during the season in the field (cold periods, drought periods, light intensities, wind, and water supply). The soil properties may also differ and vary more in the field than in the pots in the greenhouse. All these factors can affect stomatal opening and, hence, the growth and plant development. While the seedling stage took 10 days in the greenhouse, it took three months in the field, as the growth stopped during the winter due to temperatures below the base temperature for *B. orientalis* and wheat. Therefore, GDD is not the only factor affecting plant development and should always be related to the growing conditions.

We suggest future studies should address the potential role of seed bank dynamics (e.g., seed longevity, seed distribution in different soil depths, and seed predations). Various field management strategies need to be studied to reduce the soil seed bank (*e.g*., growing a perennial dense crop (grass field), fallowing). It is obvious that timely weed control in the spring (BBCH: 11–12) is essential to reduce the harmful impact of the weed on the wheat yield.

## 4. Materials and Methods

*Boreava orientalis’* plants were collected in a field in July 2017. The plants were mechanically threshed to collect seeds for the experiments. The experiments were conducted from 2018 to 2020 at Kurdistan University, Department of Agriculture and Plant Breeding. The experiments were conducted over two consecutive years under both greenhouse and field conditions. In each environment, the experimental design and replications were maintained consistently across years, and the year was considered in the statistical analysis.

The effect of light and temperature on seed germination was studied in a laboratory. Phenological development in relation to GDD was studied in a greenhouse and a field using the BBCH scale (2-digits).

### 4.1. Germination Experiment

The effect of four different light treatments on seed germination was studied by exposing the seeds to the following ratio between hours of light and darkness: 24/0; 16/8; 8/16; 0/24. For each treatment, twenty-five seeds were placed in Petri dishes on filter paper moistened with water containing 0.2% potassium nitrate. There were four replicates per treatment. The Petri dishes with seeds were placed at 4 °C for a week to break seed dormancy (stratification) and thereafter moved to a germination chamber at 20 °C and exposed to the four treatments.

### 4.2. Greenhouse Experiment

Phenological studies were conducted in a greenhouse with a minimum temperature of 20 °C (night) and a maximum temperature of 35 °C (day). One hundred plastic pots (height: 20 cm; diameter: 20 cm) were filled with a clay loam soil (500 g/pot) (Table 3) mixed with animal manure (500 g/pot) (Table 4) with sandy clods placed at the bottom of the pot for drainage. The pots were divided into four replicates of 25 pots. Before *B. orientalis* was sown, seed dormancy was broken in the laboratory, the same way as in the germination experiment. Afterwards, three seeds were sown in each pot on 18 September 2020, and the development of the plants was recorded during the experiment.

### 4.3. Field Experiment

A field with a clay loam soil (Table 3) was plowed and afterwards sown with wheat cultivar Sardari (75 kg/500 m^2^) at a 12 cm row spacing using a seeder (model Jang AP1, seed sower, South Korean) that mechanically controlled the weeds simultaneously. Before sowing, seed dormancy was broken the same way as in the other experiments. The air temperatures in the field were recorded hourly by a meteorological station. A 2 × 2 m area in the field was visited daily to record the growth stages of the *B. orientalis* and wheat plants from emergence to seed ripening. Irrigation was done once a week on average to avoid drought. We used the Zadoks scale [18] and the BBCH scale to describe the growth stages of wheat [22] and *B. orientalis*. GDD were calculated using the minimum and maximum daily air temperatures [23].

GDD = ∑ (*T_average_*) − (*T_b_*) (1)

*T_average_* is the average temperature of 24 h. The maximum and minimum temperatures were recorded daily in the greenhouse with a thermometer and in the field by a meteorological station. *T_b_* is the base temperature (4 °C) for wheat [22] and *B. orientalis* [23].

### 4.4. Statistical Analyses

Data was analyzed using SAS software (version 9.2, SAS Institute Inc., Cary, NC, USA). The appropriate statistical models included the relevant factors and their interactions. Mean comparisons were performed at the 5% probability level. Graphs and figures were generated using R software (version 4.1.0, R Core Team (2021) R: A language and environment for statistical computing. R Foundation for Statistical Computing, Vienna, Austria, https://www.R-project.org/).

## 5. Conclusions

We presented the first accurate and detailed phenological record of *B. orientalis,* including the timing of developmental stages and external morphological characteristics. This information will facilitate the exchange of new findings and collaborative research based on a shared understanding of the species. Detailed phenological observations, integrated with meteorological data, allow linking growth stages to management practices, providing a precise and practical guide for growers. The phenological stages of waxy-leaved mustard closely resemble the major stages of the wheat life cycle. The results of this study indicate that the timing of flower emergence, pollination, and seed ripening in waxy-leaved mustard overlaps with wheat development. Consequently, wheat harvesting coincides with seed dispersal and shattering of this weed, leading to increased contamination of wheat seeds. These phenological descriptions are highly valuable for both production applications and future scientific studies of *B. orientalis*. Furthermore, if similar data are collected across multiple fields over several years, they can contribute to research on the effects of global climate change.

## Figures and Tables

**Table 1:** *Boreava orientalis* growth stages (BBCH) related to Growing Degree Days (GDD) and accumulated GDD estimated in a greenhouse.

Phenological Stages	Date	Duration of Stage (Day)	GDD	Accumulated GDD	BBCH
Seedling	28 September	10	335	335	00–09
Leaves development	7 October	9	312	647	10–19
Stem elongation	25 October	28	758	1405	30–39
Buds emergence	2 November	7	265	1670	50–59
Inflorescence emergence	10 November	8	288	1958	60–69
Seed development	20 November	10	335	2293	70–79
Ripening	1–10 December	10	335	2628	80–89
Senescence					90–99

**Table 2:** *Boreava orientalis* growth stages according to Growing Degree Days (GDD) and accumulated GDD in the field.

Phenological Stages	Date	Duration of Stage (Day)	GDD	Accumulated GDD	BBCH
Seedling	10–23 December	110	1261	1261	00–09
Leaves development	22 April	13	270	1531	10–19
Stem elongation	27 April	5	46	1577	30–39
Buds’ emergence	5 May	8	116	1693	50–59
Inflorescence emergence	15 May	10	136	1829	60–69
Seed development	15 June	30	551	2380	70–79
Ripening	26 June	11	244	2624	80–89
Senescence					90–99

**Table 3:** Some physical and chemical properties of the soil used in the greenhouse and field.

Soil Texture	N (%)	P (mg/kg)	K (mg/kg)	Organic Carbon (%)	CaCO_3_ (%)	EC (dS/m)	Acidity
clay loam	0.08	9.2	198	0.07	25	2.3	7.8

**Table 4:** Average nutrient content of sheep manure (dry matter basis).

N (%)	P (%)	K (%)
2.3	1	2

## Data Availability

The original contributions presented in this study are included in the article. Further inquiries can be directed to the corresponding author.
